# Protein Kinase B2 (PKB2/AKT2) Is Essential for Host Protection in CVB3-Induced Acute Viral Myocarditis

**DOI:** 10.3390/ijms23031489

**Published:** 2022-01-27

**Authors:** So-Hee Kim, Ha-Hyeon Shin, Jin-Ho Kim, Jung-Ho Park, Eun-Seok Jeon, Byung-Kwan Lim

**Affiliations:** 1 Department of Biomedical Science, Jungwon University, Goesan-gun 28024, Korea; sohee5404@naver.com (S.-H.K.); hahyun925@naver.com (H.-H.S.); rlawlsgh3069@naver.com (J.-H.K.); 2 Bio-Evaluation Center, Korea Research Institute of Bioscience and Biotechnology, Cheongju 28116, Korea; jungho@kribb.re.kr; 3 Division of Cardiology, Samsung Medical Center, Sungkyunkwan University School of Medicine 50 Irwon dong, Gangnam-gu, Seoul 06351, Korea; eunseok.jeon@samsung.com

**Keywords:** coxsackievirus B3, protein kinase B2, toll-like-receptor4, myocarditis, innate immunity

## Abstract

Protein kinase B2 (AKT2) is involved in various cardiomyocyte signaling processes, including those important for survival and metabolism. Coxsackievirus B3 (CVB3) is one of the most common pathogens that cause myocarditis in humans. The role of AKT2 in CVB3 infection is not yet well understood. We used a cardiac-specific AKT2 knockout (KO) mouse to determine the role of AKT2 in CVB3-mediated myocarditis. CVB3 was injected intraperitoneally into wild-type (WT) and KO mice. The mice’s survival rate was recorded: survival in KO mice was significantly decreased compared with WT mice (WT vs. KO: 73.3 vs. 27.1%). Myocardial damage and inflammation were significantly increased in the hearts of KO mice compared with those of WT mice. Moreover, from surface ECG, AKT2 KO mice showed a prolonged atria and ventricle conduction time (PR interval, WT vs. KO: 47.27 ± 1.17 vs. 64.79 ± 7.17 ms). AKT2 deletion induced severe myocarditis and cardiac dysfunction due to CVB3 infection. According to real-time PCR, the mRNA level of IL-1, IL-6, and TNF-α decreased significantly in KO mice compared with WT mice on Days 5 after infection. In addition, innate immune response antiviral effectors, Type I interferon (interferon-α and β), and p62, were dramatically suppressed in the heart of KO mice. In particular, the adult cardiac myocytes isolated from the heart showed high induction of TLR4 protein in KO mice in comparison with WT. AKT2 deletion suppressed the activation of Type I interferon and p62 transcription in CVB3 infection. In cardiac myocytes, AKT2 is a key signaling molecule for the heart from damage through the activation of innate immunity during acute myocarditis.

## 1. Introduction

Coxsackievirus B3 (CVB3) is a member of the family Picornaviridae, and, along with polioviruses, it belongs to the *Enterovirus* genus. Although most enterovirus infections are subclinical, acute myocardial inflammation triggered by these infections can induce severe arrhythmias and sudden cardiac death or may lead to the development of myocarditis and dilated cardiomyopathy [1,2,3]. Myocarditis is an inflammatory disease of the myocardium, which can have various causes. The clinical features of myocarditis are polymorphic, ranging from no symptoms to cardiogenic shock and death [4,5,6,7]. Most often, myocarditis results in chronic heart failure. There is strong evidence that inhibition of the TLR4 system ameliorates myocardial inflammation in viral myocarditis [8,9,10]. Fairweather et al. reported that TLR4-deficient mice are more resistant to CVB3 infection and have decreased inflammatory responses, viral replication, and myocarditis than wild-type mice [11].

Previous reports demonstrated that the regulation of ERK and AKT cell signaling activity is important for the propagation of CVB3 in infected cells. The inhibition of both signals completely blocked CVB3 replication in HeLa cells [12]. Especially, AKT signal inhibition by a PI3K inhibitor strongly suppressed CVB3 propagation and subacute phase myocardial damage in a myocarditis mouse model [13]. AKT is an essential survival molecule with a pivotal role in regulating cardiac geometry and function [14]. The AKT family of serine-threonine kinases consists of three isoforms, AKT1, AKT2, and AKT3, each encoded by a distinct, highly conserved gene. All three isoforms are expressed in the myocardium, although AKT1 and AKT2 comprise the vast majority of total AKT protein in the heart [15,16]. AKT2 regulates glucose uptake, fatty acid transport, and glycogen synthase activity [17,18,19], but the role of AKT2 in the development of pathologic acute cardiac damage such as CVB3-induced viral myocarditis is unknown.

Here, we used an AKT2 loss-of-function murine model to assess the role of AKT2 in cardiac growth, metabolism, and cardiomyocyte survival. We demonstrated that AKT2 has important protective roles in the development of CVB3-induced myocarditis in response to a variety of provocative physiologic and pathologic stimuli. Cardiac-specific AKT2 abolition greatly increased the mortality of mice in the acute phage of CVB3 infection. It alternated with p62 reduction and compensatory induction of TLR4 due to silencing innate immune protective cytokine interferon (IFN)-α and -β in the myocardium of the AKT2-deficient mice. Conversely, we demonstrated that AKT2 is required for cardiomyocyte survival in response to CVB3-induced acute myocardial damage.

## 2. Results

### 2.1. Cardiac-Specific AKT2 Deletion

**Figure 1:**
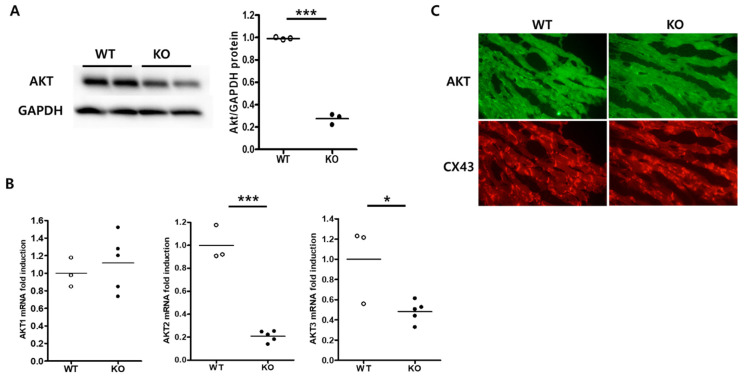
Generation of cardiac-specific AKT2 knockout mice. Protein and RNA were extracted from wild-type (WT) and AKT2 knockout (KO) mouse hearts. (**A**) Proteins were subjected to Western blot analysis using the indicated antibodies. Total AKT immunoblotting bands indicate the fold changes of total AKT normalized to GAPDH bands. (**B**) AKT1, -2, and -3 mRNA levels were determined by real-time PCR. (**C**) Heart sectioned and subjected to immunofluorescence staining. AKT (green) and connexin-43 (red) in WT and AKT2 KO hearts. All data are means ± s.e.m from three independent experiments (scale bar, 100 µm). * *p* < 0.05, *** *p* < 0.001 according to a two-tailed Student’s *t*-test.

Cardiac-specific AKT2 deletion was generated by tamoxifen administration [20]. Deletion of the *Akt2* gene was observed in the heart of wild-type (WT) and knockout (KO) mice. Total AKT protein expression was confirmed by Western blot analysis. Total AKT protein decreased in the heart of KO mice (Figure 1A). The mRNA levels of AKT1, -2, and -3 were examined by reverse transcription real-time PCR. AKT2 and AKT3 mRNA levels decreased by about 80% and 55% in KO mouse hearts compared with WT hearts, indicating that AKT2 had been silenced in the hearts of KO mice (Figure 1B). In addition, AKT2 deletion may affect AKT3 gene stability in this mouse heart. This effect could be addressed in further experiments. Transversely sectioned hearts were immunostained with antibodies against AKT and connexin-43. AKT protein decreased dramatically in the hearts of KO mice compared with those of WT mice (Figure 1C). However, we could not distinguish each isoform of the AKT proteins due to antibody issues. Thus, tamoxifen-induced gene deletion was sufficient to generate cardiac-specific AKT2 KO.

### 2.2. Changes in Mouse Survival and Heart Virus Titers in Acute Viral Myocarditis

**Figure 2:**
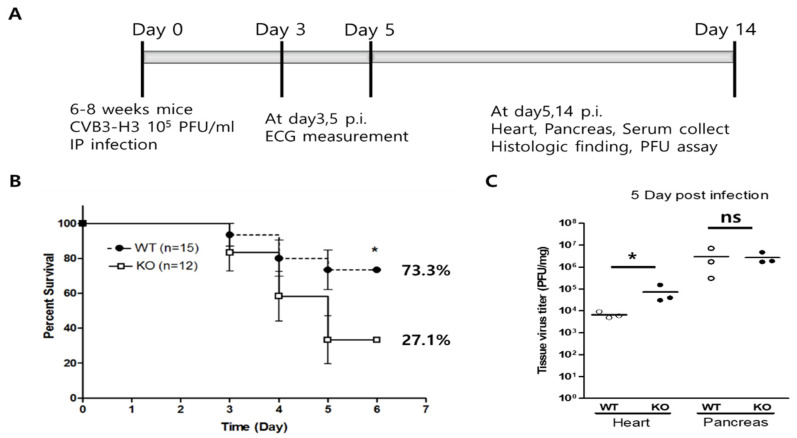
AKT2 deletion increased mouse mortality and heart virus replication. (**A**) CVB3 was injected intraperitoneally into WT and KO mice. (**B**) The survival rate of the mice was recorded on Day 14 post-infection (p.i). (**C**) The heart and pancreas were lysed on Day 5 p.i. Tissue lysates were subjected to a plaque-forming unit (PFU) assay to measure tissue virus titers. All data are the means ± s.e.m. * *p* < 0.05 according to a two-tailed Student’s *t*-test.

The effect of cardiac AKT2 deletion was examined in a CVB3-induced acute viral myocarditis model (Figure 2A). At an early stage (3–5 days post-infection), mortality dramatically increased in AKT2 KO mice compared with WT mice (72.9% vs. 26.7%, Figure 2B). On Day 5 post-infection, the virus titers were significantly higher in the hearts of AKT2 KO mice (4.54 ± 0.28 log PFU/mg of the heart) than in those of WT mice (3.82 ± 0.44 log PFU/mg of heart, *p* < 0.05), but the pancreas virus titer did not differ between them (Figure 2C). In addition, the live virus was not detected in the heart and pancreas of WT and KO mice at day14 p.i. These data indicate that AKT2 expression in cardiac myocytes is important for infected mouse survival at the subacute phase in CVB3-induced myocarditis.

### 2.3. Acute Viral Myocarditis Increases Myocardial Damage and Inflammation

**Figure 3:**
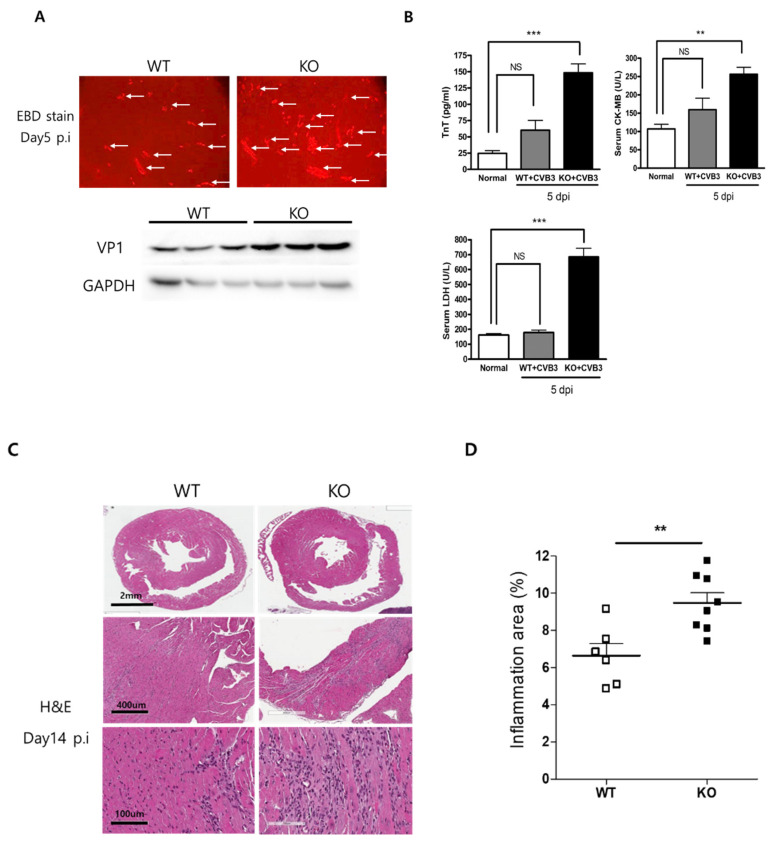
Myocardial damage increased in AKT2 knockout mice. (**A**) WT and KO mice were injected with 10% Evans blue dye (EBD) for 18 h before being sacrificed. Hearts were sectioned and subjected to fluorescence microscopy to determine the red area of EBD uptake (arrow). Heart extracts were subjected to Western blot analysis using the indicated antibodies. (**B**) Molecules indicating muscle damage (TnT, CK-MB, and LDH) were measured in Day 5 p.i mouse sera. (**C**) Histological findings using hematoxylin and eosin (H&E) staining in sectioned hearts showed inflammatory cell infiltration on Day 14 p.i. (**D**) Quantification of the inflamed area (%) of the heart (*n* = 4 each group). All data are the means ± s.e.m. ** *p* < 0.01, and *** *p* < 0.001 according to a two-tailed Student’s *t*-test.

Myocardial damage was examined by Evans blue dye (EBD) uptake and hematoxylin and eosin (H&E) staining on Day 5 post-infection. In AKT2 KO mice, the red area of EBD uptake in damaged myocardium was higher than in WT mice. Western blot analysis showed that the CVB3 VP1 capsid protein level in the heart was higher in AKT2 KO mice than in WT animals (Figure 3A). The serum levels of proteins indicating muscle damage, i.e., troponin T, CK-MB, and LDH, were significantly higher in AKT2 KO mice than in WT mice (Figure 3B). Acute myocardium damage was induced by more virus replication and infection into myocytes. Infiltration of the inflammatory cells increased significantly in AKT2 KO mouse hearts on Day 14 post-infection compared with WT mice (Figure 3C,D). The inflammatory area of the heart was quantified (inflamed area %; 6.64 ± 0.65 in WT vs. 9.48 ± 0.54 in KO, *p* < 0.01). These results indicated that AKT2 deletion increased myocyte death and myocardium damage, which increases inflammation and myocarditis.

### 2.4. Abnormal Cardiac Conduction in AKT2 KO Mice with CVB3 Infection

**Figure 4:**
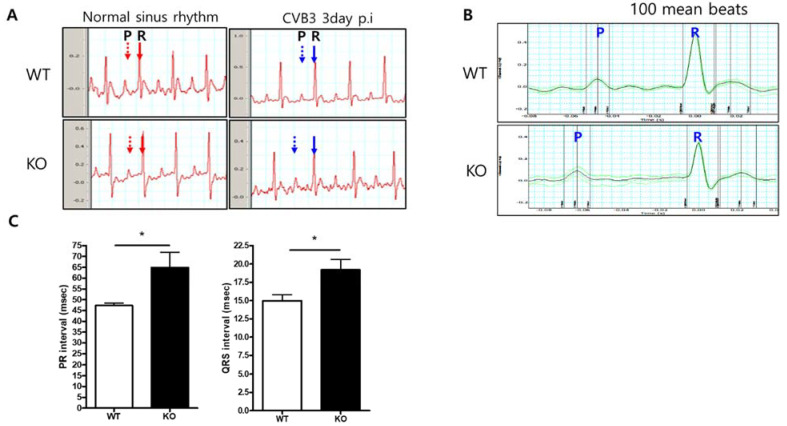
AKT2 deletion prolonged PR interval in CVB3-induced myocarditis. (**A**). Electrocardiogram (ECG) recorded on Day 3 p.i. The PR interval is extended in AKT2 KO mice (blue arrow). (**B**) ECG pattern of 100 mean beats in both groups of mice. (**C**) PR interval and QRS duration, measured by LabChart8.0 software. All data are the means ± s.e.m. * *p* < 0.05 according to a two-tailed Student’s *t*-test.

Surface electrocardiograms (ECG) were measured in 6-week-old WT (*n* = 4) and AKT2 KO (*n* = 3) mice at 3 days post-infection. The electrocardiographic abnormalities were evaluated between atrial and ventricular conduction duration (PR interval) and the ventricular conduction duration (QRS interval). Analysis of the timing and pattern of ECG demonstrated that the atrial and ventricular conduction time was delayed in AKT2 KO mice compared with WT mice. The PR interval was 47.27 ± 1.17 vs. 64.79 ± 7.17 ms (*p* < 0.05), and the QRS interval was 14.98 ± 0.81 vs. 19.19 ± 1.41 ms (*p* < 0.05) for WT and AKT2 KO mice, respectively (mean ± SD) (Figure 4). Prolonged PR and QRS intervals may not be the main cause of sudden death during the acute phase of infection. Still, this result provides strong evidence of abnormal heart function and indicates that AKT2 in the heart is important for conduction cell survival during CVB3 infection.

### 2.5. Attenuation of Acute Inflammatory Cytokine Expression

**Figure 5:**
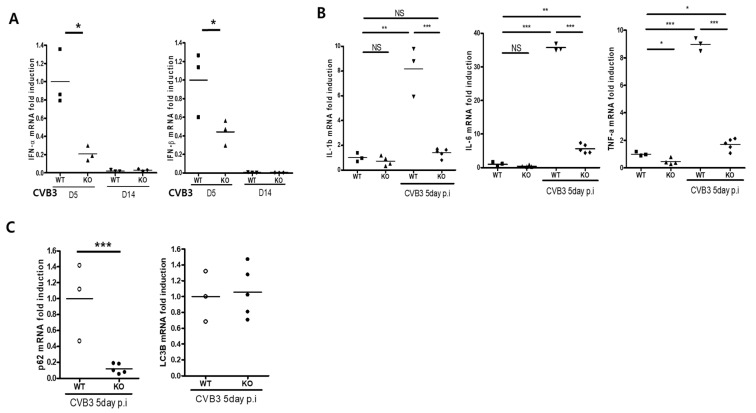
AKT2 deletion attenuates inflammatory cytokines and Type 1 interferon induction. (**A**–**C**) Total RNA was extracted from WT and KO mouse hearts on Days 5 and 14 p.i. and subjected to real-time PCR with the indicated primer sets. All data are the means ± s.e.m from two independent experiments. NS > 0.05, * *p* < 0.05, ** *p* < 0.01, and *** *p* < 0.001 according to a two-tailed Student’s *t*-test.

During CVB3 infection, inflammatory cytokine expression increases in the heart. RT-PCR analysis showed that the levels of the inflammatory cytokines interleukin-1 (IL-1), interleukin-6 (IL-6), and tumor necrosis factor-alpha (TNF-α) in the heart on Day 5 post-infection were significantly lower in the hearts of KO mice than in those of WT mice (Figure 5B). Similarly, the levels of IFN-α and IFN-β in the heart were also lower in KO mice (Figure 5A). Thus, cardiac-specific AKT2 deletion attenuated the induction of inflammatory cytokines, IFN-α, and -β in the heart under CVB3 infection. This may repress the activation of the innate immune response in acute viral myocarditis.

### 2.6. AKT2 Deletion Decreases p62 Expression in the Heart of Mice with CVB3 Infection

Autophagy induction is important for CVB3 replication in the early phase of infection. Autophagosome formation is regulated by p62 expression, which is controlled by AKT2 signaling. A previous report showed that CVB3 replication is dramatically increased by p62 disruption [21]. To determine the reason for high mortality in the acute phase of CVB3 infection, we performed real-time PCR. This analysis showed a strong reduction in the p62 mRNA level in the hearts of KO mice compared with those of WT mice on Day 5 post-infection. AKT2 deletion affected p62 expression, but this effect was not directly related to autophagy induction because the levels of LC3B had not changed at this time point (Figure 5C). In addition, no apoptosis was detected in the hearts of KO mice with acute viral myocarditis: no TUNEL positive cells were detected, and the mRNA levels of Bax, Bcl-2, and cyclinD1 were not significantly different from those in the hearts of WT mice on Day 5 post-infection (Supplemental Data Figure S1, TUNEL stain and RT-PCR). Overall, these data show that cardiac myocyte AKT2 disruption silenced p62 expression, and this induced acute damage of the heart in CVB3-infected mice, even without the induction of autophagy or apoptosis. We hypothesize that this heart damage was the main reason for the high mortality of AKT2 KO mice in the acute phase of CVB3 infection.

### 2.7. AKT2 Deletion Increases TLR4 Expression in Adult Ventricular Myocytes

**Figure 6:**
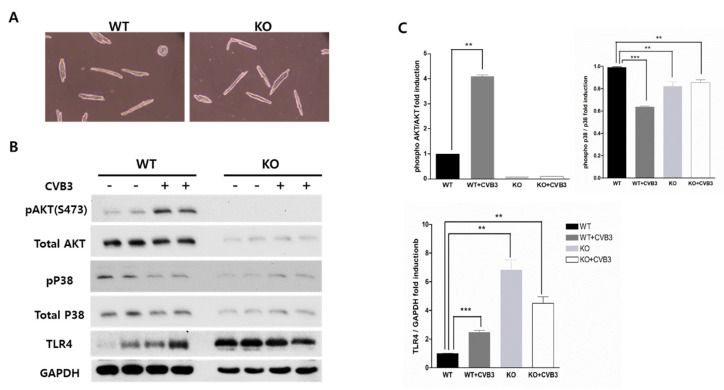
AKT2 deletion increased TLR-4 expression in isolated adult cardiomyocytes. (**A**) Adult cardiomyocytes were isolated from the hearts of WT and KO mice. Cell extracts were subjected to Western blot analysis using the indicated antibodies. (**B**,**C**) TLR-4 immunoblotting bands indicate the fold changes in TLR-4 normalized to GAPDH bands, and pAKT and pP38 bands normalized to total Akt and P38 bands. All data are the means ± s.e.m. from independent experiments. ** *p* < 0.01 and *** *p* < 0.001 according to a two-tailed Student’s *t*-test.

To obtain direct evidence of AKT2 deletion in cardiac myocytes, we cultured isolated adult ventricular myocytes from WT and KO mice. There was no difference in cell shape or viability (Figure 6A). Western blot analysis showed that CVB3 infection suppressed p38 phosphorylation in WT ventricular myocytes but increased it in KO myocytes (Figure 6B,C). However, AKT2 deletion also decreased total p38 protein expression and attenuated AKT signaling induction in CVB3 infection. Cell survival signaling could be related to activating the innate immune response and might be an early defense mechanism against CVB3 infection. Moreover, the protein expression of TLR4 significantly increased under AKT2 deletion compared with the expression levels in WT ventricular myocytes. CVB3-induced myocarditis was mediated by TLR4 overexpression in AKT KO mice (Figure 6B). Signaling through TLR4 to NF-κB and the PI3K/AKT signaling pathway may protect against myocardial damage. Cardiac AKT2 deletion may restrain this protective mechanism.

### 2.8. AKT2 Deletion Attenuates Type I Interferon Signaling in Adult Ventricular Myocytes

**Figure 7:**
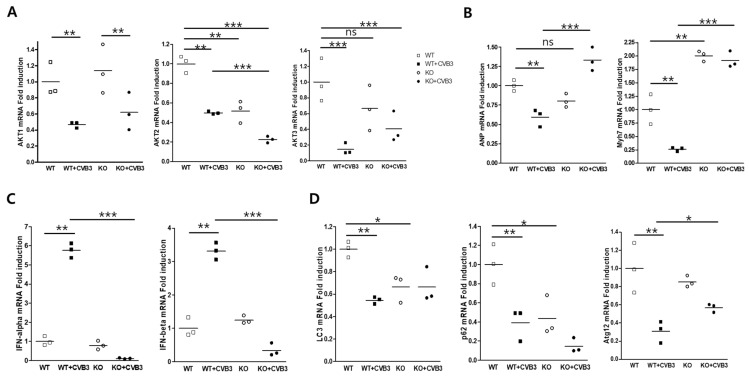
AKT2 deletion attenuated Type 1 interferon transcription during CVB3 infection. Isolated adult myocytes were infected with CVB3, and total RNA was extracted from WT and KO ventricular cardiac myocytes. RNA was subjected to real-time PCR using gene primers relating to inflammatory cytokines, heart damage, and autophagy formation. (**A**) AKT isoform expression was observed in AKT2 KO cardiac myocytes. (**B**) Cardiac myocyte damage was indicated by early gene (ANP and MyH7) induction. (**C**,**D**) Expression of Type 1 interferon and autophagosome formation genes. Expression of IFN-α, -β, and p62 was not induced by CVB3 infection in cardiac myocytes under AKT2 deletion. All data are the means ± s.e.m. from independent experiments. * *p* < 0.05, ** *p* < 0.01 and *** *p* < 0.001 according to a two-tailed Student’s *t*-test.

Gene expression in isolated adult ventricular myocytes was measured by real-time PCR. AKT1 and AKT3 mRNA expression were decreased by CVB3 infection. Moreover, AKT3 was induced by AKT2 gene deletion (Figure 7A). The levels of arial natriuretic peptide (ANP) and myosin heavy chain (Mhy7) transcripts dramatically increased in myocytes with AKT2 deletion compared with WT myocytes. Surprisingly, Type I IFN signaling during CVB3 infection was completely abrogated by AKT2 deletion (Figure 7B,C). Type I IFN is the main innate immune response molecule to activate the cellular defense mechanism in response to viral infection. The expression of genes encoding LC3B and Atg12, which are involved in autophagosome formation, was slightly induced during CVB3 infection (Figure 7D), possibly increasing virus replication. However, p62 transcription was completely knocked down by AKT2 deletion and was not stimulated by CVB3 infection. This was the main cause of the attenuation of the innate immune response and the sudden death of mice in the acute phase of infection without inflammatory cytokine induction.

## 3. Discussion

AKT is an essential survival molecule with a pivotal role in regulating cardiac geometry and function [22]. The AKT family contains three isoforms. These are usually found ubiquitously, although AKT2 is expressed in insulin-responsive tissues, including adipose tissue, skeletal muscle, the liver, and the heart [14,15,16,23]. Activating AKT signaling is essential to protect the heart during CVB3 infection by regulating virus replication and the intracellular immune response. In particular, the lack of AKT2 results in decreased expression of pro-inflammatory genes, a reduction in cell migration, and microphage M1 polarization [24,25].

CVB3 infection of the myocardium produces myocardial inflammatory cell infiltration, which is the main reason for acute and chronic heart failure [26,27]. Experimental models of viral myocarditis have suggested that the acute phase of the disease process involves both a direct viral cytopathic effect and activation of the host’s cellular immune response [28]. Innate immunity is more important for initiating the host’s first defense mechanism against acute viral infection [29]. Numerous molecules and effector cells work in concert to restrict the initial spread of an infectious focus. TNF and nitric oxide (NO) play an important role in the pathogenesis of viral myocarditis [30,31,32]. A previous report showed that the levels of inflammatory cytokines, including TNF, are elevated in patients with viral myocarditis. The mRNA and protein of those cytokines were upregulated in these patients’ hearts. However, Wada et al. [33] reported that mice with a targeted disruption of the TNF gene had increased mortality after infection with encephalomyocarditis virus compared with WT mice. The innate immune system plays an essential role as a primary sensor of invading pathogens and the induction of the host’s antimicrobial defenses [34].

In the present study, we examined the effect of cardiac-specific AKT2 deletion in CVB3-induced myocarditis. AKT2 KO mice had a high mortality rate and myocardial damage soon after CVB3 infection. However, apoptosis was not detected in CVB3-infected hearts. In addition, induction of pro-inflammatory cytokine (IL-1, IL-6, TNF) or type I IFN mRNA was not observed during CVB3 infection in AKT2 KO mice. These results indicated that the host’s innate immune response is important for sensing CVB3 infection and protecting against initial virus proliferation. AKT2 disruption in cardiac myocytes may completely abrogate the effect of the innate immune response during the acute phase of CVB3 infection. Without the innate immune response, the virus penetrates the host’s target organ more easily and induces necrotic cell death. However, activating the innate immune response during viral myocarditis may be a mixed blessing for the heart. Excessive or persistent activation of the innate immune system may lead to an exaggerated and/or chronic inflammatory process that triggers myocardial destruction and cardiac dysfunction [29,35]. In addition, AKT2 deletion may affect AKT3 gene stability in this mouse heart. This effect is not clearly explained, so this could be addressed for further experiment.

We also compared changes in the innate immune response and the genes required for autophagosome formation in isolated cardiac myocytes in the presence or absence of AKT2. We found that cardiac-specific AKT2 disruption promotes the expression of TLR4 even without CVB3 infection. TLR4 induction significantly increased the level of myocarditis 14 days after infection compared with WT mice. Zhao et al. [9] found that CVB3 can trigger TLR4 upregulation and myocarditis injury. The overexpression of TLR4 induced CVB3 replication and the production of inflammatory cytokines and chemokines. This result is consistent with previous reports [10,11]. Fairweather et al. [11] also showed that mice with defective TLR4 signaling had significantly reduced myocarditis and viral replication levels.

In addition, the results indicated that the mRNA level of the autophagy regulator p62 was dramatically decreased by AKT2 deletion. p62 is regulated by the AKT signaling pathway and may control autophagosome formation. Both p62 and AKT were directly stimulated by CVB3 infection and control virus replication. Mohamud et al. [21] reported that p62 acts as an antiviral factor and stimulates Type I IFN signaling to trigger innate immunity against enterovirus infection. Therefore, AKT2 deletion-induced silencing of p62 may attenuate inflammatory cytokine expression and early protective effects during CVB3 infection.

In conclusion, our study showed, for the first time, that AKT2 in cardiac myocytes participates in the pathogenesis of CVB3-induced myocarditis. AKT2 deletion increased the expression of TLR4 and suppression of the p62 and innate immune response. AKT2 gene silencing in cardiac myocytes induced mouse death during the acute phase of infection and increased chronic-phase cardiac inflammation and the severity of myocarditis. Thus, AKT2 signaling regulated by phosphorylation of the activation loop could control CVB3-induced viral myocarditis.

## 4. Materials and Methods

### 4.1. Viruses and Cells

Coxsackievirus B3 (CVB3) was derived from an infectious cDNA copy of the cardio trophic CVB3-H3. It was amplified in HeLa cells. Virus-infected cultured HeLa cells and mouse tissue virus titers were measured using a plaque-forming unit (PFU) assay [36]. HeLa cells were cultured in Dulbecco’s Modified Eagle’s Medium (DMEM) containing 5% fetal bovine serum and 10% penicillin-streptomycin (Wellgene Inc, Gyeongsan-si, Korea).

### 4.2. Isolation of Adult Ventricular Myocytes

The heart was perfused retrogradely for 3 min using a calcium-free buffer (heart solution) containing 120 mmol/L NaCl, 5.4 mmol/L KCl, 1.2 mmol/L MgSO4, 1.2 mmol/L NaH2PO4, 20 mmol/L NaHCO3, 5.6 mmol/L D-glucose, 20 mmol/L 2,3-butanedione monoxime (Sigma-Aldrich, St Louis, MO, USA), 5 mmol/L taurine, and 1 mmol/L pyruvate. The enzymatic digestion was achieved by perfusing a collagenase Type 2 solution (Worthington Biochemical, Lakewood, NJ, USA; final concentration: 1 mg/mL in the heart solution) at 37 °C for 10 to 20 min. Isolated myocytes were allowed to pellet by gravity in the heart solution containing 5 mg/mL bovine serum albumin. The myocytes were resuspended in a series of washing solutions with increasing concentrations of CaCl_2_ (0.125, 0.25, and 0.5 mmol/L). The myocytes were plated on laminin-precoated plates (10 mg/mL, Sigma) and cultured in a minimum essential medium (Invitrogen, Carlsbad, CA, USA) with 0.5 mmol/L CaCl_2_ for 12 h.

### 4.3. Cardiac-Specific AKT2 Knockout Mice

Cardiac-specific AKT2 KO mice were housed in the Jungwon University animal facility. Dimmers were used in the mouse cages to create twilight periods between the light and dark cycles. A room temperature between 20 and 26 °C was maintained. Mice containing the genetically modified AKT2 allele were bred with transgenic mice that harbored the cardiac-specific α–myosin heavy chain–Mer-Cre-Mer (α-MHC–MCM) transgene. Cardiac-specific AKT2 KO mice were generated by five consecutive days of tamoxifen (40 mg/kg/day) administration [20]. The study of the myocarditis model was performed in the Samsung Medical Center and Jungwon University’s animal facility. All procedures were reviewed and approved by the Institutional Animal Care and Use Committee of the Samsung Biomedical Research Institute (SBRI, #20191227002). SBRI is accredited by the Association for Assessment and Accreditation of Laboratory Animal Care International (AAALAC International) and abides by the Institute of Laboratory Animal Resources (ILAR) guide.

### 4.4. Murine Model of Coxsackievirus-Induced Viral Myocarditis

After 14 days of tamoxifen administration to induce alpha-MHC-MCM, 6-week-old AKT2 knockout (f/f Cre, KO) male and female mice and their wild-type littermates (f/f or Cre only, WT) (about 20 g in weight) were infected intraperitoneally with 2 × 10^5^ plaque-forming units (PFU) of CVB3. Mice were sacrificed on Day 5 (*n* = 20) and Day 14 (*n* = 8) post-infection, and then the heart, liver, spleen, and pancreas were harvested and analyzed. Prior to analysis, mice were injected with Evans blue dye 14 h before being sacrificed. Inflammation of the heart and myocardial damage were observed by histologic analysis.

### 4.5. Organ Virus Titers

We homogenized the hearts in Dulbecco’s Modified Eagle’s Medium (DMEM) containing 5% fetal bovine serum. The cellular debris was removed by centrifugation at 12,000 rpm for 10 min. The viral titers in the supernatants were determined by PFU assays using HeLa cells as described previously [36].

### 4.6. Reverse Transcription–Polymerase Chain Reaction

RNA was isolated from virus-infected mouse hearts. Total RNA was extracted using the TRIzol^®^ reagent (Invitrogen, Carlsbad, CA, USA) according to the manufacturer’s protocol. We synthesized complementary DNA (cDNA) using 2 g of RNA through a reverse transcription reaction using the oligo-dT primer for RNA quantification. Real-time quantitative PCR RNA or DNA analyses were performed in an ABI Sequence Detection System using the SYBR green fluorescence quantification system (Applied Biosystems, Waltham, MA, USA). The standard PCR conditions were 95 °C for 10 min, then 40 cycles at 95 °C (30 s), 55 °C (30 s), and 72 °C (30 s), followed by a standard denaturation curve. The primer sequences were as shown in Table 1.

### 4.7. Western Blot Analysis

We observed AKT and signaling protein expression levels in tissue extracted from the hearts of both WT and KO mice on Day 5 post-infection. Western blot analysis used the ECL detection system described previously. The following primary antibodies were used: anti-AKT, phospho-AKT, p38, phospho-p38, Toll-like receptor-4 (TLR4), and GAPDH from Cell Signaling Technology (Danvers, MA, USA), and CVB3-VP1 (Novocastra, Buffalo Grove, IL, USA). Protein expression levels were evaluated with Image J software (NIH; downloaded at http://rsbweb.nih.gov/ij/, accessed on 18 November 2021) based on the signal intensity of each protein using GAPDH as an internal control for protein loading.

### 4.8. Histopathology

The hearts, pancreases, and livers were fixed in 10% formalin, embedded in paraffin, and stained with hematoxylin and eosin (H&E). Trichrome staining was performed using 10-μm paraffin-embedded sections as described previously [36].

### 4.9. Surface Electrocardiograms (ECG)

For the surface ECG study, mice were anesthetized with 1% isoflurane. Needle electrodes (30 gauge) were inserted subcutaneously into the limbs. ECG signals were amplified using a PowerLab Amplifier bandpass filtered between 0.1 and 100 Hz. Data were acquired and analyzed using LabChart8.0 analysis software (ADInstruments Inc., Colorado Springs, CO, USA).

### 4.10. Statistical Analysis

The data were analyzed using Prism (GraphPad Software, San Diego, CA, USA). All data are presented as means ± standard error of the mean (SEM). The continuous variables were compared with a Student’s t-test or one-way analysis of variance (ANOVA) where appropriate. *p*-values less than 0.05 were considered statistically significant.

## Figures and Tables

**Table 1:** Real-time PCR primer sequences.

	Sense (5′→3′)	Antisense (5′→3′)
AKT1	GGA CTA CTT GCA CTC CGA GAA G	CAT AGT GGC ACC GTC CTT GAT C
AKT2	CCA ACA CCT TTG TCA TAC GCT GC	GCT TCA GAC TGT TGG CGA CCA T
AKT3	GAG ATG GAT GCG TCT ACA ACC C	TCC ACT TGC CTT CTC TCG AAC C
P62	GCT CTT CGG AAG TCA GCA AAC C	ACA GAT GGA GTC GGG AAA CTG C
ATG12	GAA GGC TGT AGG AGA CAC TCC T	GAA TCA GTC CTT TGC CCC TTC C
LC3b	GTC CTG GAC AAG ACC AAG TTC C	CCA TTC ACC AGG AGG AAG AAG G
Cyclin D1	AAC TAC CTG GAC CGC TTC CT	CCA CTT GAG CTT GTT CAC CA
Bax	TTT GCT TCA GGG TTT CAT CC	CAG TTG AAG TTG CCG TCA GA
Bcl2	AAT GAA CTC TTT CGG GAT GG	CCA ACT TGC AAT CCG ACT CA
IL-1β	TTG ACG GAC CCC AAA GAG TG	ACT CCT GTA CTC GTG GAA GA
IL-6	GTA CTC CAG AAG ACC AGA GG	TGC TGG TGA CAA CCA CGG CC
TNF-α	TTG ACC TCA GCG CTG AGT TG	CCT GTA GCC CAC GTC GTA GC
IFN-α	GCA ATG ACCATCC ATC AGC AGC T	GTG GAA GTA TGT CCT CAC AGC C
IFN-β	GCC TTT GCC ATC CAA GAG ATG C	ACA CTG TCT GCT GGT GGA GTT C
ANP	TCG TCT TGG CCT TTT GGC T	TCC AGG TGG TCT AGC AGG TTC T
Myh7	GCT GAA AGC AGA AAG AGA TTA TC	TGG AGT TCT TCT CTT CTG GAG

## Data Availability

MDPI Research Data Policies.

## Supplementary Materials

The following supporting information can be downloaded at: https://www.mdpi.com/article/10.3390/ijms23031489/s1.
