# Supplementary material for: Protein Kinase B2 (PKB2/AKT2) Is Essential for Host Protection in CVB3-Induced Acute Viral Myocarditis
Source: Int J Mol Sci. 2022 Jan 27;23(3):1489. doi: 10.3390/ijms23031489 (PMC8836114; doi:10.3390/ijms23031489)
Supplement: Supplementary file 1 [file ijms-23-01489-s001.zip › ijms-1495194-supplementary.pdf]

Supplemental data

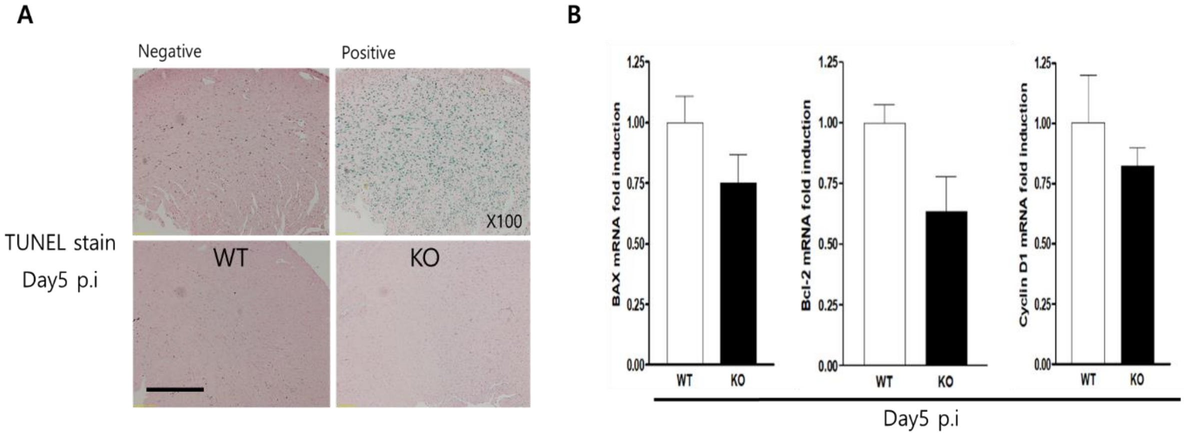


Figure S1. Effect of CAR deletion on CVB3-induced myocarditis. (A) TUNEL stain of sectioned heart show apoptosis positive myocardium using green nuclear stain. (B) Apoptotic (BAX) and anti-apoptotic (Bcl-2) gene mRNA level was performed by real-time PCR. All data are the mean ± s.e.m. from experiments performed (scale bar, 200 μm). NS>0.05 and ****P*<0.001 by two-tailed Student's *t*-test.
